# Global Analysis of Dynamical Decision-Making Models through Local Computation around the Hidden Saddle

**DOI:** 10.1371/journal.pone.0033110

**Published:** 2012-03-15

**Authors:** Laura Trotta, Eric Bullinger, Rodolphe Sepulchre

**Affiliations:** 1 Department of Electrical Engineering and Computer Science, University of Liège, Liège, Belgium; 2 GIGA Systems Biology and Chemical Biology, University of Liège, Liège, Belgium; University of Sheffield, United Kingdom

## Abstract

Bistable dynamical switches are frequently encountered in mathematical modeling of biological systems because binary decisions are at the core of many cellular processes. Bistable switches present two stable steady-states, each of them corresponding to a distinct decision. In response to a transient signal, the system can flip back and forth between these two stable steady-states, switching between both decisions. Understanding which parameters and states affect this switch between stable states may shed light on the mechanisms underlying the decision-making process. Yet, answering such a question involves analyzing the global dynamical (i.e., transient) behavior of a nonlinear, possibly high dimensional model. In this paper, we show how a local analysis at a particular equilibrium point of bistable systems is highly relevant to understand the global properties of the switching system. The local analysis is performed at the saddle point, an often disregarded equilibrium point of bistable models but which is shown to be a key ruler of the decision-making process. Results are illustrated on three previously published models of biological switches: two models of apoptosis, the programmed cell death and one model of long-term potentiation, a phenomenon underlying synaptic plasticity.

## Introduction

Decision-making processes are essential to many biological functions. At a cellular level, they are commonly implemented through bistable dynamical switches where both stable steady-states correspond to a distinct decision. Example of bistable switches are found in biological processes including cell cycle progression [1], [2], cell death signaling [3], [4], developmental processes [5], memory formation (long-term potentiation) [6], or infectious diseases such as prion propagation [7].

The paper shows how a local analysis helps understanding the global behavior of dynamical switches under assumptions that seem very plausible. The key observation is that the local analysis must not be performed around the stable steady-states of the model, which correspond to experimentally observed conditions. Rather, the local analysis is performed at a saddle point, an unstable equilibrium of the model, which is shown to be a key ruler of the (transient) decision-making process. Local analysis is shown to be particularly relevant for two biologically important analysis questions: first, the parametric robustness of the phenomenon [8] and second, which parameters influence the transient behavior, i.e the time needed to make a decision. Results are illustrated on three previously published models of bistable switches: two models of apoptosis, the programmed cell death [3], [9] and a model of long-term potentiation [6].

We argue that a local analysis at the saddle point is an excellent predictor of the global behavior and that it can save a considerable amount of time with respect to the extensive simulations required to capture the switching phenomenon under investigation.

The paper is structured as follows. The method section first illustrates the relevance of the proposed approach in two-dimensional models and describes how to extend it to models of arbitrarily large dimension. The result section then presents the results of the proposed analysis on two distinct types of published models: two models of apoptosis where the analysis is applied to an 8-dimensional [3] and a 37-dimensional [9] model, and a 10-dimensional model of long term potentiation [6].

## Methods

Bistable dynamical models have two stable equilibria. Each stable equilibrium has a distinct basin of attraction. The closure of the basins of attraction includes a common boundary that separates them. Most often, the separatrix contains an unstable saddle point, which is attractive in the separatrix but repulsive away from the separatrix. This section shows how a local analysis at this saddle point is highly relevant to understand global properties of biological switches. This is first illustrated on a two-dimensional system, then the paper describes how to extend the analysis to models of arbitrarily large dimension.

### A two-dimensional illustration

Bistability is a phenomenon that is well understood in planar models. There are many examples of two-dimensional bistable models including the famous Lotka-Volterra equations for two competing species population dynamics [10], [11], the model of genetic control proposed by Griffith [12] and the “excitatory-excitatory” (E-E) and “inhibitory-inhibitory” (I-I) models of Hopfield for neural networks [13]. In all these models, bistability is achieved thanks to the presence of a positive feedback loop, a necessary condition for bistability [14], [15]. This positive feedback results from different mechanisms of interactions such as self-induction (Lotka-Volterra model), mutual activation (Griffith and E-E models) or mutual inhibition (I-I model). As a toy example for this section, we use a model of mutual activation between two simple components:
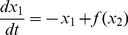
(1)

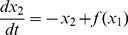
(2)


where 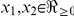 are the level of activation of two interacting components (activation of neurons, expression level of genes, concentration level of proteins,). The positive, nonlinear function 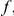 typically sigmoidal or step-like, describes the positive feedback of one component on the other. In this section, 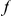 is chosen as a Hill function 
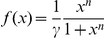
 with 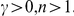 For suitable values of parameter 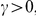 the system is bistable.

**Figure 1:**
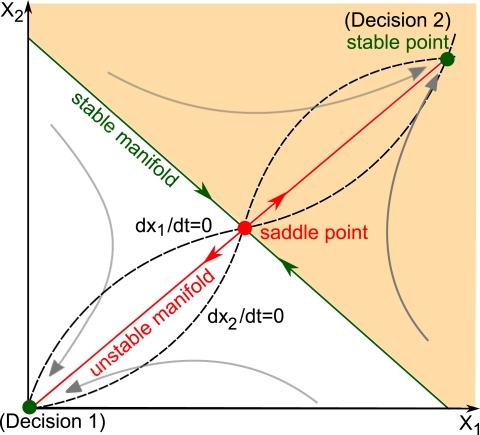
Schematic phase plane of a two-dimensional bistable model. The system has two stable steady-states (green dots) and an unstable one (red dot). These steady states lie at the intersections of the nullclines (black dashed curves), 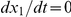 and 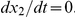 The stable manifold (green curve) of the saddle point divides the phase plane in the two basins of attraction of the stable equilibria and is therefore called a separatrix. The saddle point’s unstable manifold (red curve) connects the three equilibrium points. Any perturbation pushing the trajectory across the separatrix induces a switch in the final decision.

In addition to the two stable equilibria, two-dimensional bistable models must include a saddle point as an extra equilibrium. Figure 1 depicts the typical phase plane of a bistable model resulting from mutual activation. Equilibrium points are located at the intersection of the nullclines (black-dashed curves), i.e the curves 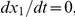 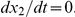 Due to the s-shape nonlinearity of nullclines (which is caused in cellular processes by specific mechanisms like ultrasensitivity [16]), the system has three equilibria. Two are stable and correspond to experimentally observable conditions (green dots): the “off” state where both 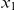 and 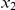 are inactivated and the “on” state where both 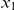 and 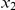 are fully activated. The third equilibrium is unstable and is therefore not seen in experiments (red dot). This point is a saddle point, i.e an equilibrium point with attractive and repulsive directions. The saddle point has a central role in the decision model: it is like a mountain pass between two valleys. Its stable manifold (green curve) divides the phase plane into the two basins of attraction of stable equilibrium points while its unstable manifold (red curve) connects the three equilibrium points.

**Figure 2:**
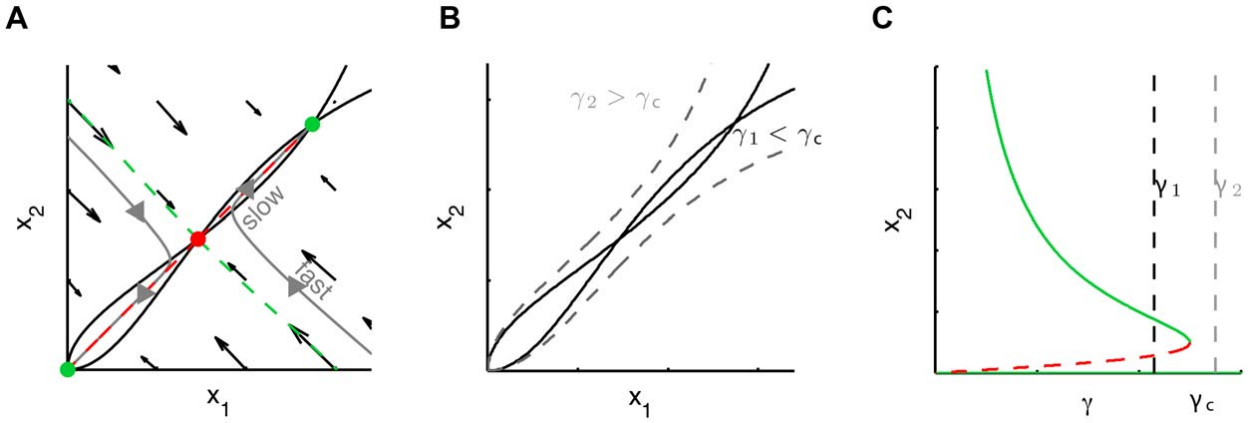
Time-scale separation at saddle point (A) and saddle node bifurcation (B-C). (A) A time-scale separation between the stable and unstable manifolds of the saddle point enhances its temporary attractivity. Trajectories (grey curves) converge to the unstable manifold (red dashed line) in the fast time-scale before sliding to a stable equilibrium point (green dot) in the slow time-scale. (B) For 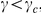 the nullclines (black curves) intersect a three equilibrium points, the system is bistable. For 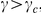 the nullclines (grey dashed curves) intersect at a simple equilibrium point, the system is monostable. (C) Corresponding diagram of bifurcation. At 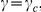 the “on” stable equilibrium branch (solid green curve) merges with the saddle branch (red dashed curve) in a so-called saddle node bifurcation.

The saddle point is a particular equilibrium point as it is both attractive and repulsive. In many bistable models, the attractivity of the saddle point is enhanced by a time scale separation at this point. Figure 2 A shows the phase portrait of a bistable model where there is a strong time-scale separation between a fast attraction to the saddle point in the stable manifold and a slow repulsion from the saddle point in the unstable manifold. This time-scale separation is visible in the vector field (black arrows) which is almost parallel to the stable manifold. Due to the time-scale separation at the saddle point, trajectories (grey curves) that start in the vicinity of the stable manifold (green-dashed line) converge in the fast time-scale to a neighborhood of the saddle point. They escape the saddle in the slow time-scale, resulting in a long transient latency. Eventually, they converge to one of the two stable equilibria. For this example, the time-scale separation persists relatively far from the stable manifold and can be observed in a large portion of the phase plane. The ratio of speeds between attractive and repulsive directions quantifies this time-scale separation. It is calculated by linearizing and computing the eigenvalues of the system at the saddle point. The positive eigenvalue 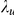 is associated with the unstable manifold while the negative eigenvalue 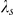 is associated with the stable one. We define the ratio
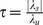
(3)


as a qualitative measure of the time-scale separation around the saddle point.

A large 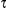 occurs in systems working close to a saddle-node bifurcation. At a saddle node bifurcation, the saddle point merges with a stable equilibrium point and the system switches from bistability to monostability. Because the positive eigenvalue 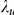 of the saddle point vanishes, the ratio 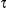 becomes arbitrarily large in its vicinity. Figure 2 B-C show how nullclines and equilibria are modified by increasing the parameter 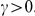 At 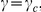 a saddle node bifurcation occurs: the system initially presenting three steady-states becomes monostable. The value of parameter 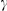 in Figure 2 A is chosen close to 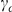 to exhibit the time-scale separation.

When 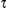 is large, a local analysis (e.g. a sensitivity analysis) of the dynamics near the saddle point reveals global properties of the bistable switch. Local analysis is routinely applied in the neighborhood of stable equilibria, which correspond to experimental steady-state conditions. In contrast, in bistable systems with a ratio 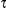 sufficiently large to observe a time-scale separation, the saddle point is very central to the system dynamics as it governs the transient behavior. This central role makes it a good point to estimate the effect of parametric perturbation on the global switching behavior. Furthermore the saddle point is also the key ruler of the bifurcation diagram in the neighborhood of the saddle node bifurcation. A perturbation that strongly affects the saddle point is thus likely to push the system beyond the bifurcation point and destroy the bistable behavior. For these reasons, a local sensitivity analysis at the saddle point is a good predictor of the global robustness of the system.

**Figure 3:**
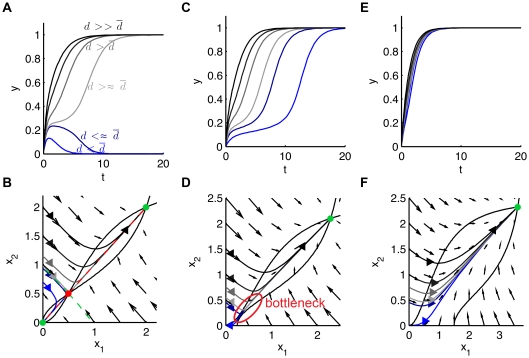
Switches with input-strength dependent delays. In Figure (A), the system is bistable. The switch occurs when the signal strength 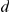 is greater than a particular threshold 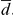 The switching time depends on 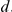 Figure (B) shows the corresponding trajectories in the phase plane. For inputs close to 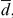 trajectories start in the vicinity of the stable manifold (green dashed curve) and converge rapidly to a close neighborhood of the saddle point (red dot). The escape from the saddle point is slow, causing the time-delay. Figures (C-D) and (E-F) show how the switch is modified by adding a production term 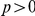 to equation (1). (C-D) 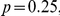 only the “off” state remains while the value of 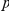 is close to the bifurcation point 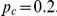 The saddle point has disappeared but its ghost creates a similar delay. One can still observe switches with input-strength dependent delays. (E-F) 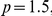 both the bottleneck and the switches with delays disappear.

A saddle point with a large 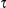 has the additional property of delaying the decision process. As an illustration, we consider an input-output version of the system (1)–(2):
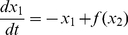
(4)

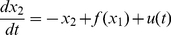
(5)

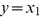
(6)


with 
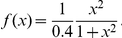
 Initially, the system is in the “off” state corresponding to decision 1. We analyzed the effect of a short duration (pulse-like) input, modeled for simplicity by a Dirac function of amplitude 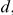 i.e 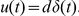 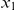 is the observed quantity. If the signal strength 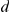 is greater than a particular threshold 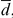 the system switches from the “off” state to the “on” state ( i.e from decision 1 to decision 2) see Figure 3 A where the output has been normalized. The “off” state corresponds to 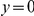 and the “on” state to 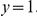 If 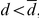 the system returns to the “off” state and no switch occurs. The switch occurs when the transient signal is strong enough to push the system state beyond the separatrix in the phase plane. Interestingly, the switching time depends on the signal strength. Figure 3 B shows the corresponding trajectories in the phase plane. The time-scale separation at saddle point forces trajectories that approach the stable manifold to rapidly converge to a neighborhood of the saddle point from which they slowly escape, causing the delay. This results in a mechanism of input-strength dependent delays with delays particularly long for inputs close to the threshold, 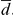


An important observation is that delayed decision making is robust to perturbations and persists beyond the bifurcation. Figures 3 A-F show how the trajectories and the phase plane are modified by adding a production term in equation (1), 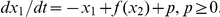 When 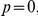 the system is bistable and one observes delays in the decision making process, see Figure 3 A-B. For 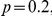 the saddle point disappears trough a saddle-node bifurcation. Despite the absence of a saddle point, see Figure 3 C-D drawn for 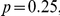 one still observes the time-delayed decision. The ghost saddle point creates a bottleneck, a well-known phenomenon [17]. This phenomenon disappears as the system moves further away from the bifurcation point, see Figure 3 E-F where 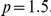


### Local analysis of a n-dimensional model

Bistability is also observed in models of dimension 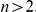 This section shows how to find and identify a saddle point in a high-dimensional model. It also describes how to extend the ratio 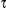 introduced for two-dimensional systems and how to compute a local sensitivity at this point.

#### Localization of the saddle point

Localizing steady states in a high-dimensional system of nonlinear differential equations is not a straightforward task because it requires finding the roots of the algebraic equation f(x) = 0. The peformance of numerical root finding algorithms is usually local, that is, roots are easily found numerically provided that a good initial guess is known. For stable steady-states of a bistable system, a few simulations of the differential equation are sufficient to provide good initial guesses since simulations will converge to one of the two stable equilibria. In a similar way, simulations initialized in the vicinity of the stable manifold of the saddle point will have a long transient near the saddle point, especially if there is a strong time-scale separation, thereby providing good initial guess for the root finding algorithm. Because the stable manifold of the saddle is a separatrix of the two basins of attraction, initializing a simulation near the stable manifold is achieved by picking up a state variable that clearly distinguishes the two stable states (this choice is often suggested by biology) and by applying a bisection procedure to identify an initial condition close to the separatrix. In this paper, we used Matlab’s ode15s for simulating the differential equations and Levenberg-Marquardt option in the fsolve algorithm for solving the algebraic equations.

#### Local stability analysis at the saddle point

The local stability of an equilibrium point, 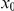 is computed by linearizing the differential equation around that point to obtain the Jacobian matrix
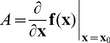
(7)


and calculating eigenvalues and corresponding eigenvectors of 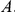 A saddle point has eigenvalues with both positive and negative real parts. ln this paper, we assume for simplicity that the saddle point is hyperbolic, i.e it has no eigenvalues with zero real part. We also assume that the linearization presents a simple positive eigenvalue 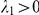 and 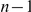 eigenvalues with a negative real part 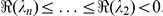 From the stable manifold theorem [18], the eigenvector associated with the positive eigenvalue 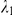 provides the tangent approximation of the unstable manifold at the saddle point while the remaining eigenvectors span an hyperplane tangent to the stable manifold. We generalize the two-dimensional definition of the ratio (3) by defining 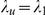 and 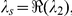 a high ratio meaning a strong time-scale separation.

#### Local sensitivity at the saddle point

Sensitivity analysis is a standard tool to quantify the effect of parameter variation on the system behavior. Local sensitivity analysis is routinely applied around stable fixed points. Here, we propose to compute the local sensitivity analysis at the saddle point. For hyperbolic steady states, the sensitivity at the steady state 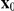 is given by
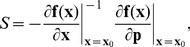
(8)


or, its normalized version for a steady state 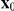 with nonzero entries
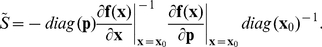
(9)


To convert this matrix into a scalar measure, we use the cumulated sensitivity of a given parameter defined as
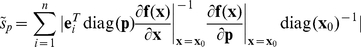
(10)


where 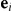 is the i-th Euclidean basis vector. The scalar quantity 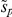 is the matrix one-norm of 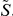


### Non local sensitivity analyses

Non local sensitivity analyses are based on numerical tools and have been used to estimate the parametric robustness of several biological systems [19]–[23]. Such methods include bifurcation diagrams [19], [20] or extensive numerical simulations such as Monte Carlo-based methods [21], [22], [24]. A limitation of these methods is that the computational task becomes considerable as the dimension of the model increases. In this paper, the results of a local sensitivity analysis at saddle point are compared with the results of a non local single parameter robustness analysis, the DOR analysis. This method consists in computing for each parameter, a *degree of robustness* (DOR) and is inspired by the method of Ma and Iglesias [20] proposed to study the robustness of oscillators. The DOR of a bistable model with respect to a particular parameter 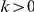 (all remaining parameters being fixed) is defined by:
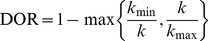
(11)


where 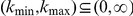 denotes the range of bistability. This global sensitivity measure is computed around (and therefore dependent on) a nominal set of parameters. A degree close to one means that the system is very robust to parameter 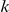 and a degree close to zero means that it is very sensitive to this parameter. The computation of the range of bistability for each parameter variation is of course a computationally demanding task. For the Eißing and Aslam models and the set of nominal parameters proposed by original authors (see Table S1 and Table S3), it is computed by drawing a diagram of bifurcation for each parameter 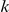 with the software XPPAUT [25]. For the 37-dimensional and highly nonlinear Schliemann model, calculating diagrams of bifurcation becomes very demanding. Instead, the interval of bistability is computed using simulations, i.e by perturbing one parameter at a time and checking that the system is still bistable. The set of parameters used for this model is referenced in Table S2.

## Results

This section presents the proposed local analysis on three published models of deterministic biological bistable systems. The analysis is first applied to a small model of the apoptotic switch proposed by Eißing et al. [3], then to a larger model of the apoptotic switch by Schliemann et al. [9] and finally to a model of long term potentiation proposed by Aslam et al. [6]. In these three models, the switch is triggered by a transient signal (pulse-like). The local analysis sheds light on the mechanism governing the switch between stable steady-states and is used to quantify the robustness of the process to parametric perturbations. The results are compared with results from non local analyses.

### Local analysis at the saddle point of apoptosis models provides global understanding of the decision-making process

Apoptosis, the predominant form of programmed cell death, is used by multicellular organisms to remove superfluous, damaged or potentially harmful cells [26]. In this process, a pro-apoptotic signal triggers a biochemical signaling cascade activating specific proteases, the initiator caspases, which then activate other proteases, the effector caspases, leading to cellular death [26]. See [27] for an overview of the broad variety of apoptotic signaling models. Among these models, several involve a feedback loop between initiator and effector caspases leading for suitable parameters to a bistable system. This is the case for the 8-dimensional model of Eißing [3] and the 37-dimensional model of Schliemann [9].

#### Eißing model

**Figure 4:**
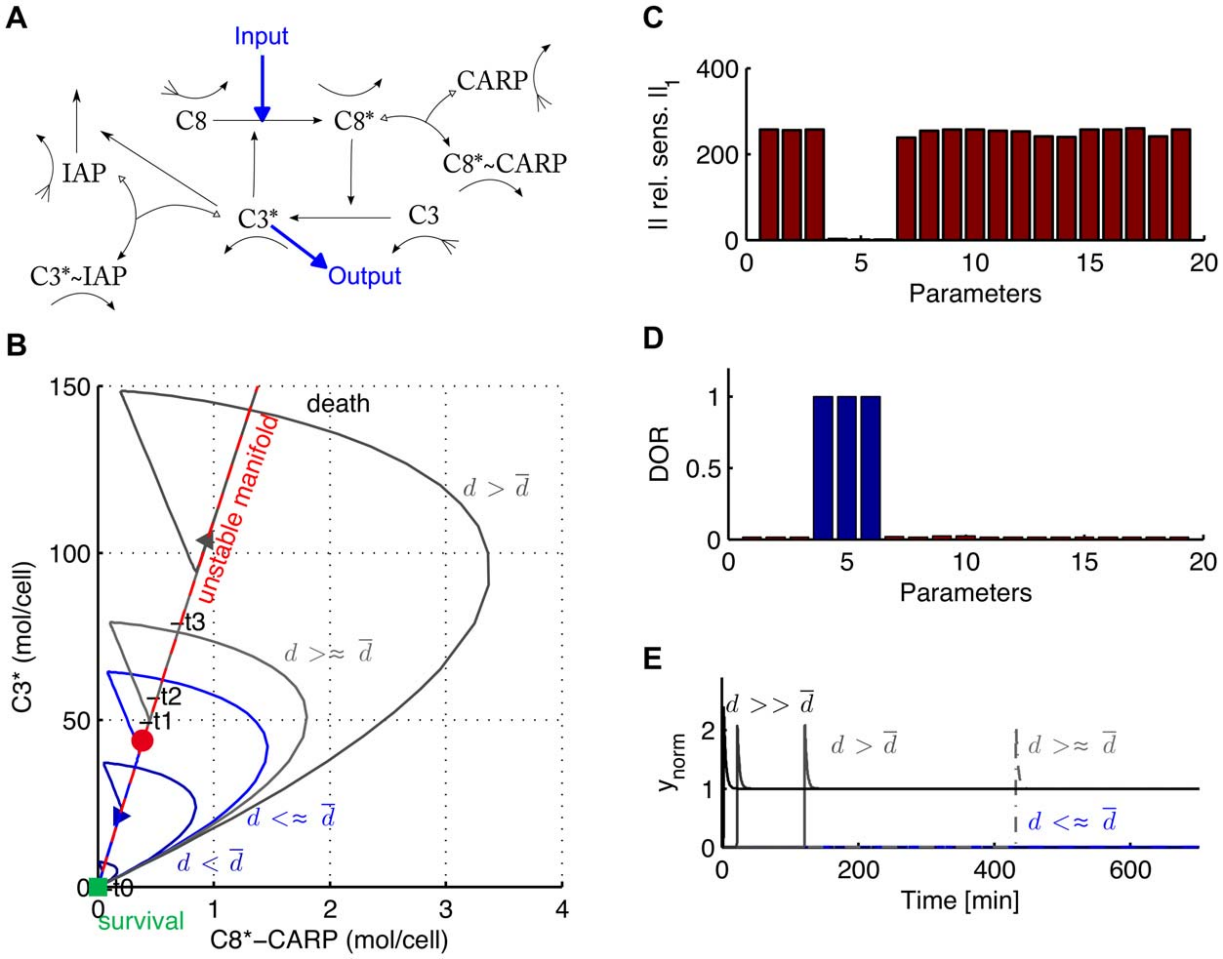
Results of the analysis of the model of apoptosis proposed by Eißing et al. [3]. (A) Model description. In response to a pro-apoptotic input signal, initiator caspases C8 become activated and activate the effector caspase C3. Activated C3, C3*, activate C8 in return through a positive feedback loop. Inhibitors CARP and IAP bind to C8* and C3* to prevent apoptosis. (B) Time-scale separation at saddle point. Trajectories rapidly converge to the unstable manifold (red dashed line) of the saddle point (red dot) and then slowly escape to reach either the life (green square) or the death steady-states. For the grey trajectory, equally distributed time markers are depicted (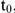 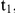 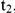 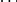) showing how trajectories are delayed in the vicinity of the saddle point. (C) Sum of relative sensitivities at saddle point. The saddle point is insensitive to parameters 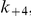 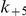 and 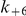 (see Supplementary table 1). These parameters have a high degree of robustness (D). (E) Output trajectories for increasing input. For input above the threshold, the system switches to the unexcited state, see the corresponding trajectories in the phase plane (B). Trajectories have been normalized such that the output equals zero in the unexcited state and equals one in the exited state. Depending on the input strength, the switch is more or less delayed. By observing trajectories in the phase plane (D), one can see that trajectories starting close to the stable manifold of the saddle point fast converge in the neighborhood of the saddle point where there are delayed before converging to the excited state creating a mechanism of delayed decision making.

The model of Eißing [3] is a model of 8 ordinary differential equations with 19 kinetic parameters, where the activation of the initiator caspase C8 is enhanced through a positive feedback loop with the effector caspase C3, see Figure 4 A. The model also involves two inhibitors of apoptosis IAP and CARP that can link to caspases to avoid apoptosis.

Eißing et al proposed an input-ouput version of the model. The input affects the concentration of activated initiator caspases 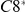 while the ouput was related to the concentration of activated effector caspases 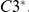 In the present analysis, the input signal directly acts on the number of initiator caspases that become activated (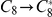) rather than an extra inflow of active initiator caspases. This slight modification with respect to [3] has been chosen to better describes the effect of a pro-apoptotic signal but the same results hold for the original input. For nominal parameter values (see Table S1), the system exhibits three steady-states with non-negative concentrations, two stable points corresponding to life and death and a saddle point.

The system linearized at the saddle point has one real positive eigenvalue 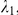 and 7 negative ones 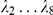 with 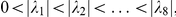 see Table 1. The ratio, 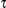 between the slowest negative eigenvalue and the positive one is high (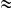
10) and reflects the high time scale separation at the saddle point. Although the model is eight-dimensional, the strong time-scale separation forces trajectories to rapidly converge to the vicinity of the saddle point before slowly escaping along its unstable manifold to asymptotically reach one of the two stable equilibria, see Figure 4 B. The cumulative sensitivity 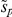 shows that the saddle is insensitive to the parameters 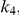 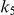 and 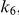 the ones controlling the degradation of free activated caspases C3* and C8* and the active degradation of an inhibitor IAP by C3*, see Figure 4 C. In Figure 4 D, this local sensitivity analysis is compared with the result of a non local robustness analysis. This analysis shows that the system is particularly robust to the parameters 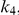 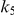 and 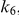 thus to the ones with low sensitivities at the saddle point. Conversely, the bistability is not robust to parameters with high sensitivities. The good match between both analyses reveals the predictive power of the local sensitivity analysis at the saddle point to estimate the robustness of the bistable behavior. Interestingly, the three insensitive parameters control the degradation of free caspases suggesting that free caspases are not involved in the death decision making process. Instead, the slow dynamics at the saddle point are mostly governed by inhibitors.

**Table 1:** Model of Eißing: Eigenvalues and ratio 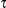.

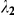	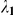	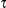
–0.0011	1.08e–04	9.9897

The presence of a saddle point with a large 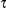 induces a delayed decision mechanism in the Eißing model. We simulated the system for inputs of increasing amplitude by modifying the initial concentrations of 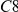 and 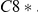 For small stimuli, the systems returns to the life state but strong stimuli induce a fast transition to the death equilibrium. By bisection, we obtained the input’s switching threshold as 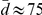 mol/cell. For inputs close to this value, the states remain relatively close to the saddle point for a long time, see Figure 4 B,E. The switching time depends on the stimulus strength.

#### Schliemann model

**Figure 5:**
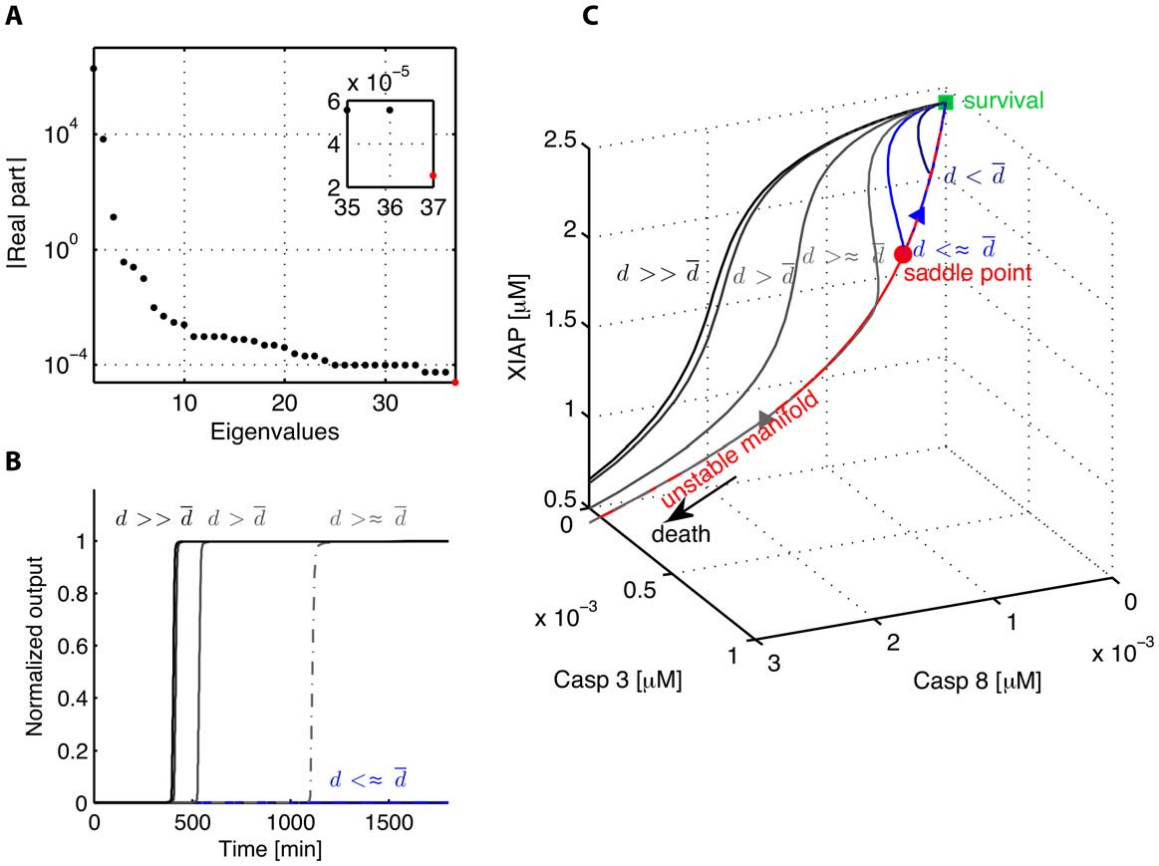
Results of the analysis of the model of Schliemann et al. [9]. (A) Magnitude of the real part of the eigenvalues of the Jacobian matrix at the saddle point, the stable ones are depicted in black while the unstable one is depicted in red. The zooms in on the three slowest eigenvalues. (B) Output trajectories for impulse inputs, slightly below (light blue solid curve), slightly above (dark grey dashed curve), above (dark grey solid curve) and significantly above (black solid curve) the decision making threshold, 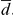 (C) Corresponding trajectories in the phase plane. Trajectories passing close to the saddle point are delayed. Trajectories follow the unstable manifold of the saddle point (red dashed curve) before reaching the survival or death state.

The model by Schliemann et al. is a much larger model of apoptosis signaling [9]. This model describes the pro- and anti-apoptotic signaling pathways induced by the stimulation with the cytokine TNF. On the one hand, TNF enhances the activity of NF-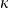
B, an important transcription factor for anti-apoptotic proteins. On the other, TNF internalizes and then activates the initiator caspase Caspase 8, which is part of a positive feedback loop of mutual activation of Caspase 8, Caspase 3 and Caspase 6. In the input-output version of the system, the input modifies the initial concentration of TNF while the output is chosen as the concentration of activated Caspase 3. For nominal parameter values (see Table S2), the model has a total of 37 states and is also bistable with a saddle point having only one positive eigenvalue, which furthermore is the smallest one in absolute values, see Figure 5 A and Table 2. The ratio 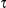 is less pronounced here, approximatively a factor two. This is still enough to delay the switch for impulse inputs close to the input threshold, see Figure 5 B for simulations with various input intensities around the threshold level 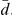 The delayed decision making is particularly pronounced for inputs slightly above the threshold, where the delay is quite significant (about one day). Visualizing the trajectories in the state space illustrates the importance of the saddle point and of its unstable manifold (Figure 5 C). Inputs close to the transition threshold result in trajectories that first converge to the proximity of the saddle point before diverging along the unstable manifold. Because of a smaller 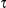 value than in the model of Eißing, the convergence is less pronounced for inputs not very close to the threshold.

**Table 2:** Model of Schliemann: Eigenvalues and ratio 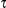.

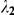	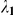	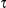
–5.6e–05	2.6e–05	2.2

**Figure 6:**
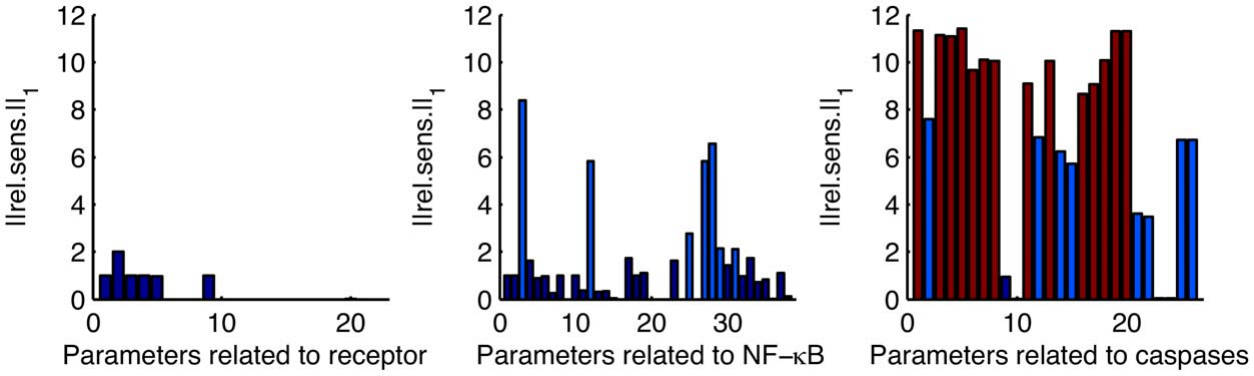
Sensitivity analysis at saddle point for the model of apoptosis proposed by Schliemann et al. [9]. The parameters have been divided in three sets. The first one include the parameters controlling the reactions involving the binding of TNF to receptor, the second one the parameters controlling the activity of NF-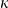
B and the last one the parameters linked to the reactions governing caspases and their inhibitors.

Figure 6 shows the relative sensitivities at saddle point. It should be noted that the sum of relative sensitivities is computed over the states with a non-zero concentration. Interestingly, the linearized system is sensitive to the parameters controlling the reactions which involve the caspases and their inhibitors while it is quite robust to parameters controlling the reactions that govern the binding of the ligand to the receptor. This suggests an essential role for caspases and inhibitors in the control of the switch from life to death in agreement with a recent analysis of the system based on experimental data [28]. Red parameters, i.e parameters with a large sensitivity at saddle point have a 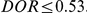 At the opposite, dark blue parameters, i.e the parameters with a low sensitivity at saddle point, have a 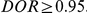 As for the model of Eißing, sensitivity analysis at saddle point is a good predictor of the robustness of the bistable behavior.

### Model of long-term potentiation

**Figure 7:**
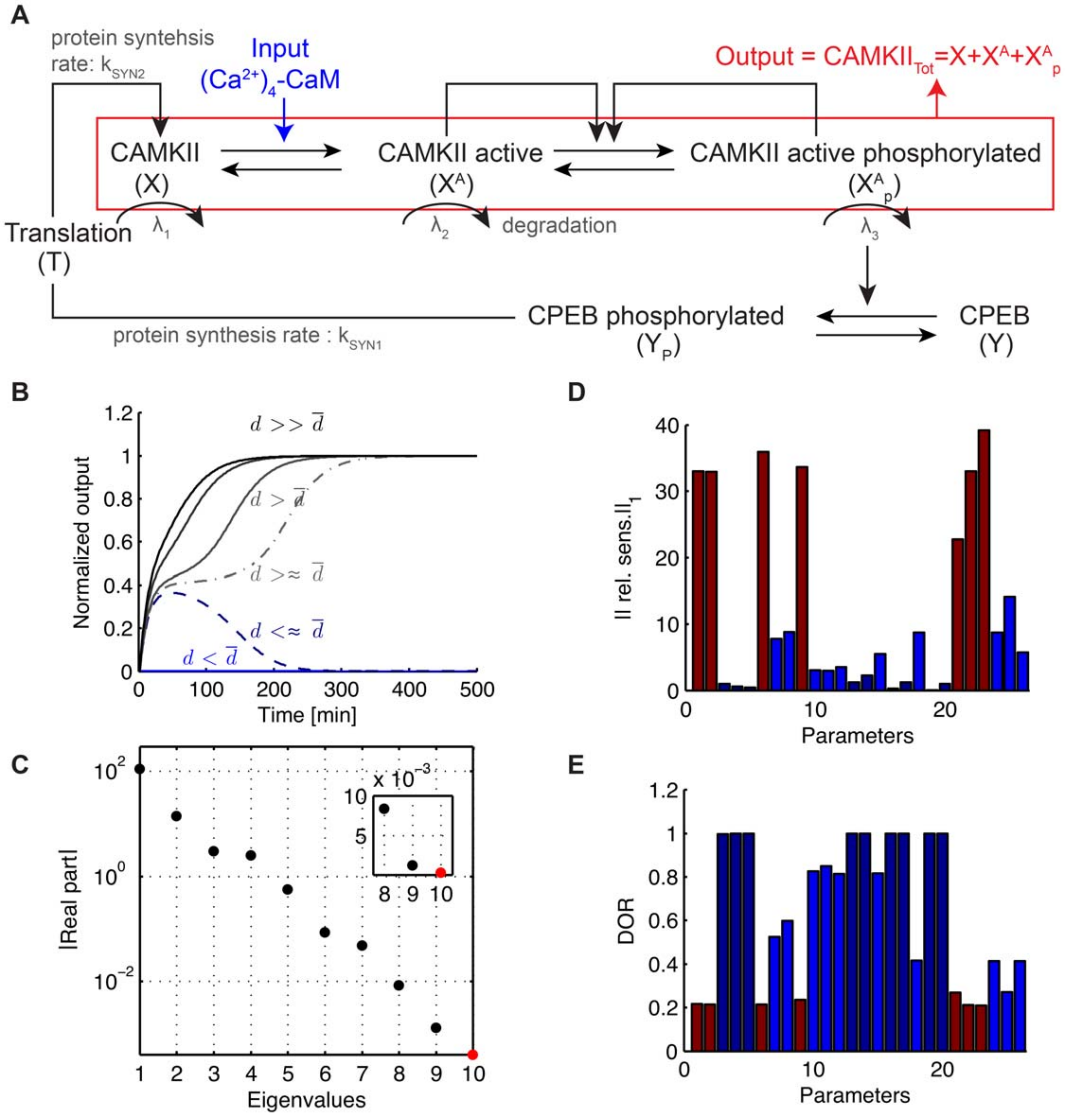
Model of Aslam et al. [6]. (A) The model describes the positive feedback loop between the protein 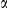-CaMKII and the translation factor CPEB1. The protein 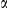-CaMKII can be in one of three states: inactive (X), active (X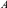) and phosphorylated (X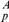). When active and phosphorylated, 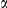-CaMKII phosphorylates CPBE1 which in turn can initiate the translation of a new 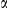-CaMKII protein [6]. (B) Trajectories for increasing inputs showing the delay close to the threshold 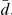 (C) Magnitude of the real part of the eigenvalues of the Jacobian matrix at the saddle point, the stable ones are depicted in black while the unstable one is depicted in red. The inlet zooms in on the three slowest eigenvalues. (D) Sensitivity at saddle point and (E) degrees of robustness (DOR). Parameters with a high sensitivity (red) have a low degree of robustness. Conversely, parameters with a low sensitivity (dark blue) have a high DOR.

This section shows the results of our local analysis to a model of long term potentiation proposed by Aslam et al. [6]. Long term potentiation (LTP) describes the long-lasting increase in synaptic strength described in learning and memory processes [29]. Aslam et al. proposed a model of late LTP (L-LTP) in agreement with experimental data where long term potentiation is achieved thanks to the presence of a bistable switch resulting from the molecular loop between the kinase (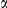-CaMKII) and the translation regulation factor (CPEB1), see Figure 7 A. The protein 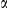-CaMKII can be in one of three states: inactive (X), active (X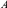) and phosphorylated (X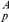). When active and phosphorylated 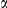-CaMKII phosphorylates CPBE1 which in return initiates the translation of a new 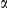-CaMKII protein creating a positive feedback leading to a fast increase of the total concentration of 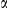-CaMII. For biologically plausible parameters values (see Table S3), the 10-dimensional ODE model is bistable. The induction of L-LTP is modeled by a brief pulse (10 seconds) which transiently increases the basal level of (Ca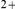)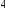-CaM. For weak pulses, the system returns to the initial steady state corresponding to low concentration of total CaMKII. For stronger pulses, the system switches to the other stable steady state and the total concentration of CaMKII increases to approximately twice its basal level, see figure 7 B.

**Figure 8:**
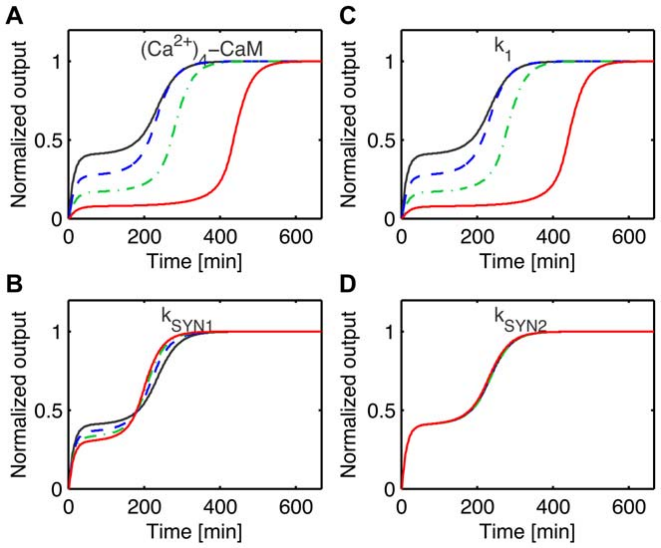
Parameter perturbation of the model of Aslam et al. [6]. (A)-(D) The switching is depicted for nominal values of the parameters (black curve), 10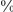 of parameter perturbation (blue dashed curve), 20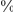 (green dashed-doted curve) and 30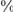 of variation (red curve). The system is simulated for an input slightly above the threshold, i.e 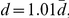 where the threshold 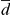 is recomputed for each parameter perturbation. (A) (Ca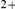)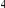-CaM (parameter 22), (B) k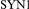 (parameter 18), (C) k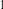 (parameter 1) and (D) k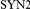 (parameter 20). Both the switching threshold and time are affected by perturbation of parameters (Ca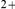)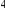-CaM and k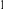. In contrast, the switching threshold and time are insensitive to a perturbation of parameter kSYN2.

**Figure 9:**
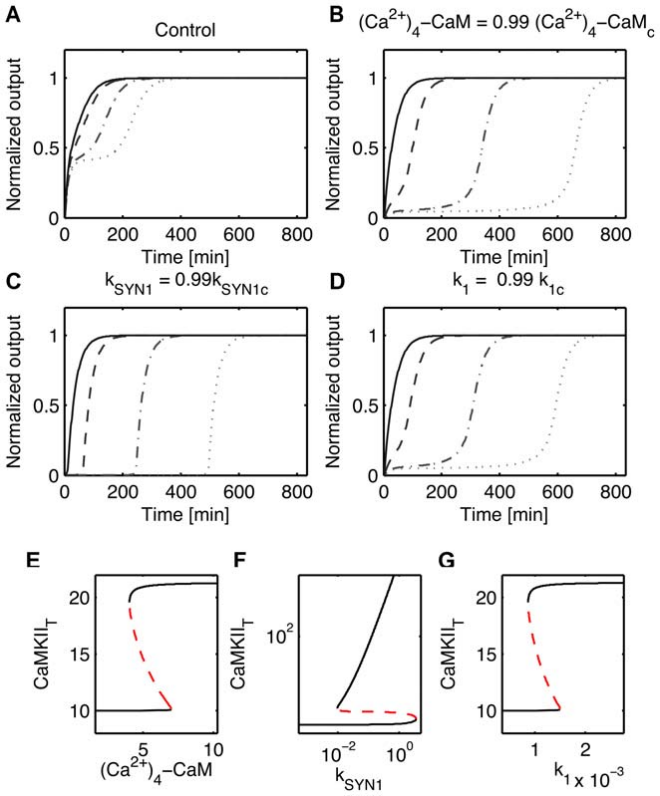
Influence of the distance to a bifurcation on the switching for the model of Aslam et al. [6]. (A)-(D) Switching responses for the Aslam model for different input intensities 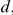 above the threshold 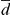: 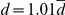 (doted line), 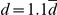 (dashed-doted line), 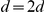 (dashed line), 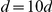 (solid line). (A) Nominal model. (B)-(D) Perturbed models with single parameter set to 0.99 of its upper bifurcation value. (E)-(G) Bifurcation diagrams for the parameters 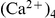-CaM, k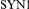 and k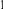. (B) and (E) 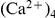-CaM, (C) and (F) 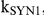 and (D) and (G) k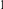.

We numerically found a saddle point and computed the eigenvalues of the Jacobian matrix at this point, see Figure 7 C and Table 3. All the eigenvalues are real with 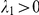 and 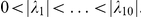 As for the models of Eißing and Schliemann, the unstable manifold of the saddle point is one-dimensional. The ratio 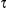 is smaller than for the model of Eißing but it is sufficient to induce a time-scale separation at saddle point and observe delays in the switch see Figure 7 B. The local sensitivity analysis at the saddle point correlates well with the degree of robustness: parameters with high sensitivities have a low degree of robustness while parameters with low sensitivities have a high degree of robustness, see Figure 7 D-E where the relative sensitivities have been computed for the non-zero parameters of the nominal model. In addition, we looked at the effect of a parametric perturbation on the switch. We chose a set of parameters with different sensitivities at saddle point and perturbed one parameter at a time. Then we computed the new switching threshold 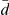 and simulate the system for increasing inputs above this new threshold 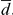 Simulations show that both the switching threshold and the delay durations are mostly affected by the sensitive parameters at the saddle point, such as the basal level of (Ca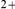)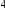-CaM (parameter 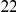) and the rate of activation of CAMKII k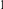 (parameter 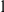), see Figure 8. In contrast, the switch was insensitive to modification of the protein synthesis rate kSYN2 (parameter 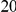) as predicted by the local sensitivity analysis at the saddle. As previously illustrated in dimension 2, delayed decision making is enhanced close to a saddle node bifurcation where the ratio 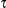 is generally high. This is illustrated by modifying the initial value (control) of parameters (Ca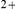)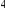-CaM, kSYN1, k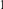 and putting them close to their value at bifurcation, (Ca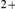)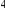-CaM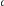, kSYN1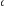, k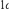, see Figure 9.

**Table 3:** Model of Aslam: Eigenvalues and ratio 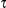.

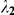	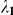	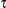
–0.0013	4.04e–04	3.3

## Discussion

### Local analysis for global predictions

Sensitivity analysis is routinely applied to systems linearized around a stable equilibrium point in order to test the parametric robustness of the model. In this paper, we propose to study the sensitivity around an unstable equilibrium point to analyze the parametric robustness of a bistable decision process. Performing a local analysis around an unstable equilibrium point may seem of little relevance since it does not correspond to an experimental condition. However, it was shown that the saddle point is a key ruler of the transient behavior of bistable decision processes, especially in the case of strong time-scale separation.

Our approach is based on the hypothesis that the system has a saddle point with a ratio 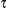 which is large enough to induce a time-scale separation between a fast attraction to the saddle in the stable manifold and a slow repulsion from the saddle in the unstable manifold. Every model satisfies the required hypothesis in the vicinity of a saddle node bifurcation as the real part of the positive eigenvalue 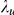 vanishes close to the saddle node bifurcation. The phenomenon is therefore commonly encountered in bistable models and it is not surprising to observe large 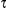 in models of biological switches.

The two global predictions derived from the local analysis rely on the time-scale separation in the following sense: (i) the use of 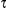 to predict decision delays is a direct consequence of considering the unstable manifold of the saddle as a valid one-dimensional reduction of the full model, see [30] for a formal reduction based on singular perturbation theory. (ii) The use of local sensitivity analysis to predict the robustness of bistability is particularly relevant when the nominal set of parameters is chosen close to a saddle node bifurcation, which implies a strong time-scale separation at the saddle point.

We analyzed three previously published models of bistable switches and compared our results with results of non local methods such as diagrams of bifurcation and numerical simulations. For the three models, results of the local sensitivity analysis are excellent predictors of the results obtained with the non local methods. Local sensitivity analysis allowed us to identify the parametric perturbations that are the most likely to destroy the switch. In particular, in both models of apoptosis and for the set of nominal parameters proposed by original authors, the apoptotic switch is particularly sensitive to the parameters controlling the reactions involving caspases linked to inhibitors. This result is in agreement with previous analyses of the models which identified the complexes caspases-inhibitors as key rulers of the decision making process [31], [32].

Our analysis also reveals a simple mechanism to create switches with delays. This type of behavior has been observed in several biological switches including apoptosis [33], [34]. In apoptosis, experimental results have shown the existence of a variable latent period before the fast activation of effector caspases [33], [34]. Recent experiments suggest that the variability in the duration of the latent period has a non-genetic origin and depends on the protein levels in the cell [35]. These results are well captured by the proposed mechanism where the variability of the delay depends on the initial concentration of enzymes involved in the death process and the way trajectories are attracted and then repulsed by the saddle point.

### Decision-making in non bistable models

A striking feature of the proposed analysis is that it captures important properties of bistable switches models beyond the blanket hypotheses of the paper, i.e the assumption of a saddle point with strong time-scale separation and of two stable points. This is because, on the one hand, time-scale separation is a robust phenomenon even for moderate values of 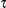 and, on the other hand, because of the ghost effect of the saddle point beyond the bifurcation. In that sense, the debate whether bistability is a necessary feature of the decision making processes is irrelevant to the results of the paper. For this reason, predictions made with our analysis in the vicinity of a saddle-node bifurcation apply beyond the bifurcation, that is, to models that are monostable and contain no saddle.

### Delayed decision-making and decision reversibility

The mechanism of delayed decision making has strong biological relevance because it is related to potential reversibility. In state space, the long latency period of delayed decision takes place close to the separatrix of the basins of attraction. As a consequence, small perturbations have the ability to revert the switch during the entire latency period. This potential of reversibility might be particularly relevant for the long term potentiation model of Aslam. The importance of the model lies in its ability to reproduce experimental results, in particular to account for the different effects of applying inhibitors during the induction or the maintenance phase of L-LTP: if applied during the induction of L-LTP, protein synthesis inhibitors can block L-LTP but they do not reverse the potentiation when applied during the maintenance phase of L-LTP [29], [36]. Moreover blocking the 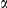
CaMKII activity stops the L-LTP induction phase but not the maintenance phase [37], [38]. These observations are completely consistent with our explanation that small perturbations can revert the decision during the latency period, i.e close to the saddle point in state space, but not once the system has reached one of the two equilibria.

### Future directions

The proposed analysis is purely deterministic. However, in real organisms, the decision-making process is affected by noise [39], [40]. Noise can affect the dynamics in many ways but clearly affects both the probability of switching and the time required to make a decision. An interesting extension of this work would be the analysis of stochastic systems in order to determine in which measure the local analysis remains a good predictor under the presence of noise. In this paper, we only studied models of decision-making at the intracellular level. However, bistability has been used to describe decision-making in other contexts such as collective decision in neuronal populations and insects colonies [41], [42]. Further work should determine what further insight could be gained from applying the proposed methodology to these systems.

## Supporting Information

Table S1
**Parameters for the model of Eißing, all in 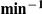
** **[3].**
(PDF)

Table S2 **Parameters for the model of Schliemann** **[9].**
(PDF)

Table S3 **Parameters for the model of Aslam** **[6].**
(PDF)
