# Supplementary material for: Global Analysis of Dynamical Decision-Making Models through Local Computation around the Hidden Saddle
Source: PLoS One. 2012 Mar 15;7(3):e33110. doi: 10.1371/journal.pone.0033110 (PMC3305308; doi:10.1371/journal.pone.0033110)
Supplement: Table S1 — Parameters for the model of Eißing, all in [3]. (PDF) [file pone.0033110.s001.pdf]

1	$k_{+1} = 5.8\text{e-}5$
2	$k_{+2} = 1\text{e-}5$
3	$k_{+3} = 0.0005$
4	$k_{+4} = 0.0003$
5	$k_{+5} = 0.0058$
6	$k_{+6} = 0.0058$
7	$k_{+7} = 0.0173$
8	$k_{+8} = 0.0116$
9	$k_{+9} = 0.0039$
10	$k_{+10} = 0.0039$
11	$k_{+11} = 0.0005$
12	$k_{+12} = 0.001$
13	$k_{+13} = 1.16\text{e-}2$
14	$k_{-3} = 0.21$
15	$k_{-8} = 464$
16	$k_{-9} = 507$
17	$k_{-10} = 81.9$
18	$k_{-11} = 0.21$
19	$k_{-12} = 40$
