# Supplementary material for: Global Analysis of Dynamical Decision-Making Models through Local Computation around the Hidden Saddle
Source: PLoS One. 2012 Mar 15;7(3):e33110. doi: 10.1371/journal.pone.0033110 (PMC3305308; doi:10.1371/journal.pone.0033110)
Supplement: Table S2 — Parameters for the model of Schliemann [9]. (PDF) [file pone.0033110.s002.pdf]

	Parameters related to receptor	Parameters related to NF-κB	Parameters related to caspases and inhibitors
1	$ka_1 = 0.001$ (s^{-1})	$ka_{21} = $ 2e-05 (μMs^{-1})	$ka_{24} = $ 2.413e-04 (μMs^{-1})
2	$ka_2 = $ 3.226e-07 (μMs^{-1})	$kd_{21} = $ 1e-04 (s^{-1})	$kd_{24} = $ 1e-04 (s^{-1})
3	$ka_3 = $ 2.352e-02 (s^{-1})	$ka_{22} = $ 3e-06 (μMs^{-1})	$ka_{55} = $ 1e-04 (μMs^{-1})
4	$ka_4 = $ 5e-05 (μMs^{-1})	$kd_{22} = $ 1e-04 (s^{-1})	$kd_{55} = $ 1e-04 (s^{-1})
5	$kd_4 = $ 1e-04 (s^{-1})	$ka_{23} = $ 7.028e-07(μMs^{-1})	$ka_{56} = $ 2.5e-05 (μMs^{-1})
6	$ka_5 = $ 2.352e-02 (s^{-1})	$kd_{23} = $ 1e-04 (s^{-1})	$kd_{56} = $ 1e-04 (s^{-1})
7	$ka_6 = $ 5.6e-05 (s^{-1})	$ka_{25} = $ 3e-06 (μMs^{-1})	$ka_{57} = $ 2e-07 (μMs^{-1})
8	$ka_7 = $ 5.6e-05 (s^{-1})	$kd_{25} = $ 1e-04(s^{-1})	$kd_{57} = $ 1e-04 (s^{-1})
9	$ka_8 = $ 5.6e-05 (s^{-1})	$ka_{26} = $ 1e-04 (s^{-1})	$ka_{58} = $ 1e-04 (s^{-1})
10	$ka_9 = $ 5.6e-05 (s^{-1})	$ka_{27} = $ 1e-04 (s^{-1})	$ka_{59} = $ 1e-04 (s^{-1})
11	$ka_{10} = 12600$ (μM^{-1}s^{-1})	$ka_{28} = $ 3.3e-05 (s^{-1})	$ka_{60} = $ 1e-04 (s^{-1})
12	$kd_{10} = $ 1.134e-05 (s^{-1})	$ka_{29} = $ 3.3e-04 (s^{-1})	$ka_{61} = $ 1e-04 (s^{-1})
13	$ka_{11} = 0.17$ (μM^{-1}s^{-1})	$ka_{30} = $ 1e-04 (s^{-1})	$ka_{62} = $ 9.446e-07 (μMs^{-1})
14	$ka_{12} = $ 5e-04 (s^{-1})	$ka_{31} = $ 3.3e-05 (s^{-1})	$kd_{62} = $ 1e-04 (s^{-1})
15	$ka_{13} = $ 5e-04 (s^{-1})	$ka_{32} = $ 3.3e-05 (s^{-1})	$ka_{63} = $ 1e-04 (s^{-1})
16	$ka_{14} = $ 5e-04 (s^{-1})	$ka_{33} = $ 1e-01(s^{-1})	$ka_{64} = 0.05$ (μM^{-1}s^{-1})
17	$ka_{15} = 0.01$ (s^{-1})	$ka_{34} = $ 3.3e-04 (s^{-1})	$ka_{65} = 0.05$ (μM^{-1}s^{-1})
18	$ka_{16} = $ 5.3216e+04 (μM^{-2}s^{-1})	$ka_{35} = $ 3.3e-04 (s^{-1})	$ka_{66} = 0.05$ (μM^{-1}s^{-1})
19	$ka_{17} = $ 1e+05 (μM^{-2}s^{-1})	$ka_{36} = $ 3.3e-04 (s^{-1})	$ka_{67} = $ 1.2e+06 (μM^{-1}s^{-1})
20	$ka_{18} = 4$ (μM^{-1}s^{-1})	$ka_{37} = 50$ (μM^{-1}s^{-1})	$kd_{67} = 600$ (s^{-1})
21	$ka_{19} = 1000$ (μM^{-2}s^{-1})	$ka_{38} = 0.02$ (s^{-1})	$ka_{68} = 6$ (μM^{-1}s^{-1})
22	$ka_{20} = 2$ (μM^{-1}s^{-1})	$ka_{39} = 3.45$ (μM^{-1}s^{-1})	$ka_{69} = $ 5e-05 (s^{-1})
23		$ka_{40} = 0.2$ (μM^{-1}s^{-1})	$ka_{70} = 0.5$ (μM^{-1}s^{-1})
24		$ka_{41} = 10$ (μM^{-1}s^{-1})	$ka_{71} = 0.5$ (μM^{-1}s^{-1})
25		$ka_{42} = $ 1.67e-04 (s^{-1})	$ka_{72} = $ 1e+06 (μM^{-1}s^{-1})
26		$ka_{43} = 1$ (μM^{-1}s^{-1})	$kd_{72} = 600$ (s^{-1})
27		$ka_{44} = $ 5e-07(s^{-1})	
28		$ka_{45} = 0.8$(s^{-1})	
29		$ka_{46} = $ 2.5e-03(s^{-1})	
30		$kd_{46} = $ 5e-04(s^{-1})	
31		$ka_{47} = 0.1$ (μM^{-1}s^{-1})	
32		$ka_{48} = 0.001$ (s^{-1})	
33		$ka_{49} = $ 2.5e-06 (s^{-1})	
34		$ka_{50} = 0.5$ (s^{-1})	
35		$ka_{51} = $ 3.333e-07 (s^{-1})	
36		$ka_{52} = $ 6.25e-02 (s^{-1})	
37		$ka_{53} = $ 4.4e-07 (s^{-1})	
38		$ka_{54} = $ 3.275e-02 (s^{-1})	
