# Supplementary material for: Global Analysis of Dynamical Decision-Making Models through Local Computation around the Hidden Saddle
Source: PLoS One. 2012 Mar 15;7(3):e33110. doi: 10.1371/journal.pone.0033110 (PMC3305308; doi:10.1371/journal.pone.0033110)
Supplement: Table S3 — Parameters for the model of Aslam [6]. (PDF) [file pone.0033110.s003.pdf]

1	$k_1 = 1.106\text{e-}03$ (μM^{-1}s^{-1})
2	$k_{-1} = 14$ (s^{-1})
3	$k_2 = 0.0224$(μM^{-1}s^{-1})
4	$k_{-2} = 0.028$ (s^{-1})
5	$k_3 = 0.021$ (s^{-1})
6	$k_4 = 1.4$ (μM^{-1}s^{-1})
7	$k_{-4} = 1.7063\text{e-}02$ (s^{-1})
8	$k_5 = 0.0616$ (s^{-1})
9	$k_6 = 2.7496$ (μM^{-1}s^{-1})
10	$k_{-6} = 0.28$ (s^{-1})
11	$k_7 = 2.786$ (s^{-1})
12	$k_8 = 0.028$(μM^{-1}s^{-1})
13	$k_{-8} = 0.14$ (s^{-1})
14	$k_9 = 0.2520$ (s^{-1})
15	$k_{10} = 2.772$(μM^{-1}s^{-1})
16	$k_{-10} = 0.14$ (s^{-1})
17	$k_{11} = 2.38$ (s^{-1})
18	kSYN1= 1.624e-02 (μM^{-1}s^{-1})
19	$k_{-12} = 0.28$ (s^{-1})
20	k$SYN2 = 112$ (s^{-1})
21	BASAL = 10 (μM)
22	(Ca^{2+})$_4$-CaM (basal level) = 5.144 (AU)
23	P = 2.214e-03 (μM)
24	T = 0.05 (μM)
25	$\lambda_1 = \lambda_2 = \lambda_3 = 7.28\text{e-}04$ (s^{-1})
26	YT = 10 (μM)
27	BASAL1= 0 (μM)
28	BASAL2 = 0 (μM)
